# Tripeptidyl Peptidase 1 (TPP1) Deficiency in a 36-Year-Old Patient with Cerebellar-Extrapyramidal Syndrome and Dilated Cardiomyopathy

**DOI:** 10.3390/life12010003

**Published:** 2021-12-21

**Authors:** Agnieszka Ługowska, Joanna K. Purzycka-Olewiecka, Rafał Płoski, Grażyna Truszkowska, Maciej Pronicki, Paulina Felczak, Mateusz Śpiewak, Aleksandra Podlecka-Piętowska, Martyna Sitek, Zofia T. Bilińska, Przemysław Leszek, Małgorzata Bednarska-Makaruk

**Affiliations:** 1 Department of Genetics, Institute of Psychiatry and Neurology, Al. Sobieskiego 9, 02-957 Warsaw, Poland; jkpurzycka@yahoo.com (J.K.P.-O.); makaruk@ipin.edu.pl (M.B.-M.); 2 Department of Medical Genetics, Medical University of Warsaw, ul. A. Pawińskiego 3c, 02-106 Warsaw, Poland; rploski@wp.pl; 3 Molecular Biology Laboratory, Department of Medical Biology, National Institute of Cardiology, ul. Alpejska 42, 04-628 Warsaw, Poland; gtruszkowska@ikard.pl; 4 Department of Pathology, The Children’s Memorial Health Institute, al. Dzieci Polskich 20, 04-730 Warsaw, Poland; m.pronicki@czd.pl; 5 Department of Neuropathology, Institute of Psychiatry and Neurology, Al. Sobieskiego 9, 02-957 Warsaw, Poland; pfelczak@ipin.edu.pl; 6 Magnetic Resonance Unit, Department of Radiology, National Institute of Cardiology, ul. Alpejska 42, 04-628 Warsaw, Poland; mspiewak@ikard.pl; 7 Department of Neurology, Medical University of Warsaw, ul. Banacha 1a, 02-097 Warsaw, Poland; apodlecka@wum.edu.pl (A.P.-P.); neurologia1-sekretariat@wum.edu.pl (M.S.); 8 Unit for Screening Studies in Inherited Cardiovascular Diseases, National Institute of Cardiology, ul. Alpejska 42, 04-628 Warsaw, Poland; zbilinska@ikard.pl; 9 Department of Heart Failure and Transplantology, National Institute of Cardiology, ul. Alpejska 42, 04-628 Warsaw, Poland; przemyslaw.leszek@ikard.pl

**Keywords:** tripeptidyl peptidase 1 (TPP1), ceroid lipofuscinosis 2 (CLN2), autosomal recessive spinocerebellar ataxia type 7 (SCAR7), dilated cardiomyopathy, cerebellar ataxia

## Abstract

We report on a 36-year-old man with cerebellar-extrapyramidal syndrome and severe heart failure because of dilated cardiomyopathy of unknown origin. Dysarthria and cardiac arrhythmia began at early childhood (4 years of age). Brain MRI (28 years of age) demonstrated severe cerebellar atrophy. At the age 32, he presented with dysarthria, ataxia, dystonia, and tremor of the right hand, bilateral slowed neural conduction in the visual pathways, and decreased mental acuity. At the age of 33 years, the patient underwent cardiac transplantation because of severe dilated cardiomyopathy. In the *TPP1* gene, biallelic variants were identified: previously reported p.(Leu13Pro) and novel p.(Tyr508Cys) variant. Additionally, hemizygous novel missense variant in the *ABCD1* gene was inherited from the mother p.(Arg17His). Normal very-long-chain fatty acids (VLCFA) levels both in patient and his mother excluded *ABCD1* mutation as the pathogenic one. Tripeptidyl peptidase 1 (TPP1) activity was reduced (8,8 U/mg protein/h; reference range: 47.4 ± 10.7). In light microscopy the biopsy specimens obtained from explanted heart showed severe myocyte hypertrophy with perinuclear vacuolization with inclusions. Electron microscopy revealed absence of lipofuscin accumulation, no ultrastructural curvilinear profiles, fingerprint bodies, or granular osmiophilic deposits (GRODs) in lysosomes. As described here, the patient presents clinical symptoms observed in benign forms of ceroid lipofuscinosis type 2 (CLN2) and simultaneously some features of autosomal recessive spinocerebellar ataxia type 7 (SCAR7), which is also caused by mutations in the *TPP1* gene.

## 1. Introduction

The neuronal ceroid-lipofuscinoses (NCLs) are a group of neurodegenerative, inherited lysosomal storage disorders of variable disease course, and age of onset ranging from early childhood to adolescence. Typical clinical features are epilepsy, vision loss, and motor and cognitive function deterioration, leading to early death. So far, 14 types of NCLs have been distinguished.

NCL of an unknown form was firstly described in 1826 by Dr. Otto Christian Stengel [1]. Patients with late infantile onset NCL were first reported by Jansky in 1908 and Bielschowsky in 1913 [2,3].

The deficiency of tripeptidyl peptidase 1 (TPP1) activity leads to accumulation of autofluorescent storage material (AFSM), which is composed of different lipids and proteins [4]. AFSM is observed in lysosomes, which are the main cellular organelles involved in degradation of high molecular compounds. TPP1 (EC 3.4.14.9) is one of lysosomal hydrolases comprising 365 amino acids. It is a serine-carboxyl peptidase, that sequentially removes N-terminal tripeptides from small peptides. It also displays a minor endoprotease activity [5]. The lack of active TPP1 is the biochemical cause of neuronal ceroid lipofuscinosis (NCL) type 2 (CLN2), a neurometabolic disorder inherited in an autosomal recessive trait. CLN2 belongs to rare diseases with a frequency ranging from 6–8 cases per 100,000 live births [6]. CLN2 is a result of pathogenic mutations (variants) in the *TPP1* gene, located on chromosome 11p15.4 and comprising 13 exons.

In CLN2, first clinical signs and symptoms appear at the age of 2–4.5 years and include seizures and myoclonic ataxia as well as speech delay, followed by developmental milestones loss and psychomotor decline, visual impairment leading to blindness, and death in the childhood or early teenage years [7,8,9]. The most common, classic form of CLN2–LINCL presents with early onset (2–4.5 years) of ataxia or seizures and progressive loss of motor function, as well as cognitive decline followed by visual impairment, resulting in death in the second decade of life [10].

Diagnostically important symptoms include progressive cortical–subcortical atrophy, especially of cerebellum and cerebrum, accompanied with an enlargement of lateral ventricles, normal basal ganglia and thalami observed in MRI, photoparoxysmal response in EEG, and curvilinear bodies in tissue biopsies. Pathological changes of the “bull’s eye” type can be present in the optical coherence tomography (OCT) [11].

Homozygous or compound heterozygous pathogenic mutations in the *TPP1* gene lead to different forms of CLN2 and influence the phenotype. Among over 100 pathogenic variants in the *TPP1* gene, the two most frequent mutations—namely, c.622 C > T (p.(Arg208*) and c.509–1 G > C—lead to the classic form of CLN2.

Chen et al. reported that 60.8% of mutations in the *TPP1* gene result in late infantile neuronal ceroid lipofuscinosis (LINCL) most severe phenotype [10]. There is a genotype–phenotype correlation indicating that mutations leading to amino acid chain truncation or invariant splice-site mutations result in complete TPP1 loss of function and haploinsufficiency [10]. Only 5.2% of mutations in the *TPP1* gene lead to the juvenile phenotype of CLN2. Among them are mainly missense mutations (resulting in variable residual TPP1 activity) and variant splice-site mutations (leading to aberrant or normal transcripts) [12]. In the juvenile form of CLN2, the first symptoms usually appear at 6–10 years of age and they include vision loss, dementia, epilepsy, and motor function deterioration. Typically, protracted disease course leads to death in the mid-20s of 30s of life. Other rare atypical forms of CLN2 have also been described [10,13,14,15].

Only 1% of mutations in the *TPP1* gene express clinically as a very rare disease-spinocerebellar ataxia 7 (SCAR7), which usually starts in childhood or adolescence with cerebellar ataxia and pyramidal manifestations; and deep sensory, motor, and neurocognitive impairment, with variable severity but generally of more benign character than classic CLN2 [16,17,18]. So far, in patients with SCAR7, epilepsy, ophthalmological changes, or cognitive regression characteristic of NCL have not been yet described. On the other hand, SCAR7 patients do not display optic atrophy, deafness, scoliosis, diabetes, fundus abnormalities and cardiac involvement, described as typical for other ataxias [18], and the progression of clinical symptoms is slow.

In 2013, Sun et al. identified *TPP1* as the causative gene for SCAR7. To date, seven patients with SCAR7 from three families were reported [16,17]. The absence of ultra-structural curvilinear profiles in tissue biopsies is different from the known CLN2 phenotypes.

It was suggested that loss of function variants abolishing TPP1 enzyme activity lead to CLN2 disease, whereas variants that diminish TPP1 enzyme activity lead to slowly progressive SCAR7. SCAR7 is caused by compound heterozygous variants in *TPP1* gene. Probably, partial deficiency of the TPP1 enzyme explains the mild phenotype [16,17] and supraventricular tachycardia. At 36 years of age, the patient presented with cerebellar-extrapyramidal syndrome and dilated cardiomyopathy.

## 2. Materials and Methods

### 2.1. Patient

The boy was born as the first child to healthy, young, non-consanguineous parents. His two younger sisters are healthy. As a newborn and in infancy he developed normally until the age of 4 years, when he developed dysarthria (speech disturbances and stuttering) and cardiac arrhythmia (paroxysmal supraventricular tachycardia) concomitantly.

**Figure 1:**
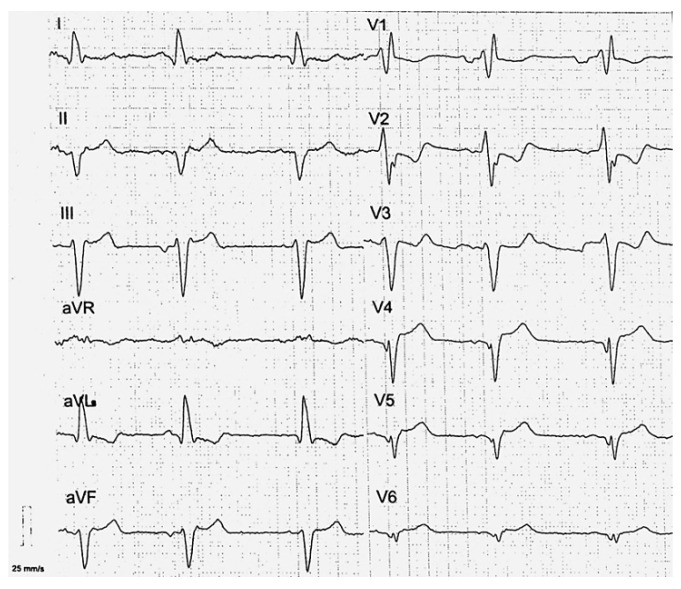
Standard 12-lead electrocardiogram (ECG). Paper speed—25 mm/sec. Voltage gain—10 mm/mV. Sinus bradycardia 54 beats per minute. Sinistrogram (−65 degrees). The PR interval is the time from the onset of the P wave to the start of the QRS complex, it reflects conduction through the atrio-ventricular node, equals to 124 ms (normal value 120–200ms). The QRS complex is the major positive deflection on the ECG, produced by ventricular depolarization and is prolonged up to 182 ms (normal value 60–110 ms). Left anterior fascicular block is recognized on the basis of sinistrogram (−65 degrees). qR complexes in leads I, aVL, and rS complexes in leads II, III, and aVF are visible. Right bundle branch block is present with QRS duration > 120ms and rSR’ QRS pattern in lead V1 (“M-shaped” QRS complex). In addition, regression of r waves in leads V3–V6 is present that corresponds well with wall thinning of lateral segments on cardiac magnetic resonance.

**Figure 2:**
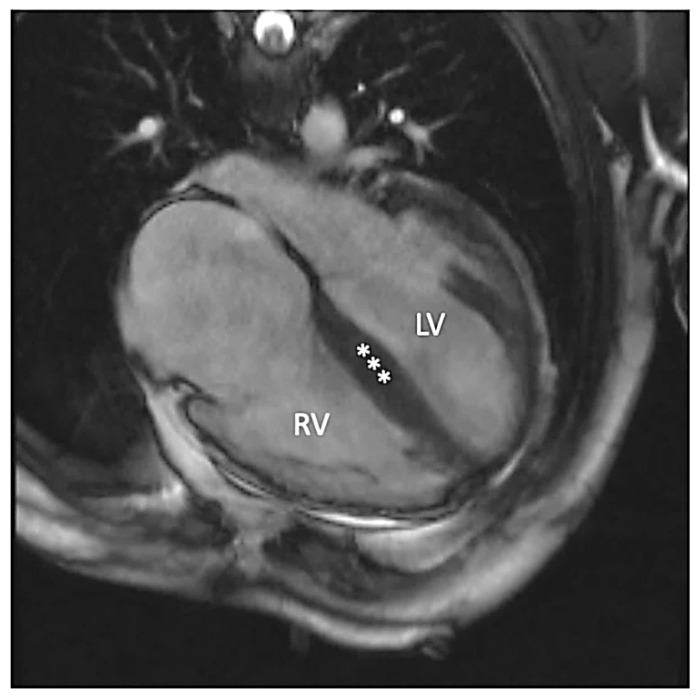
Steady-state free precession four-chamber image demonstrating enlarged left (LV) and right (RV) ventricles. Additionally, increased myocardial thickness in the septal segments (asterisks) and wall thinning of lateral segments of the left ventricle are seen.

**Figure 3:**
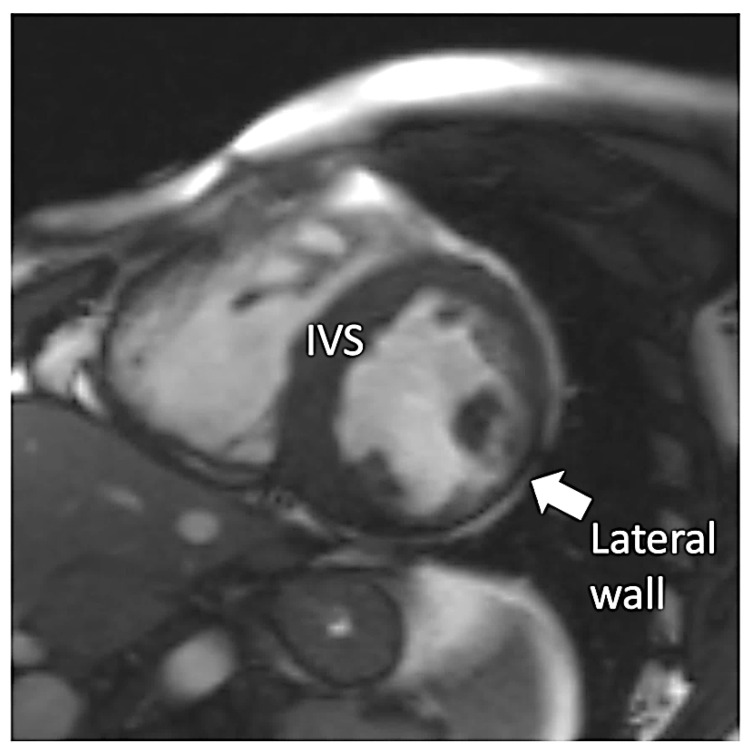
Steady-state free precession short axis view demonstrating increased thickness of the interventricular septum (IVS) and thinning of the left ventricular lateral wall (arrow).

**Figure 4:**
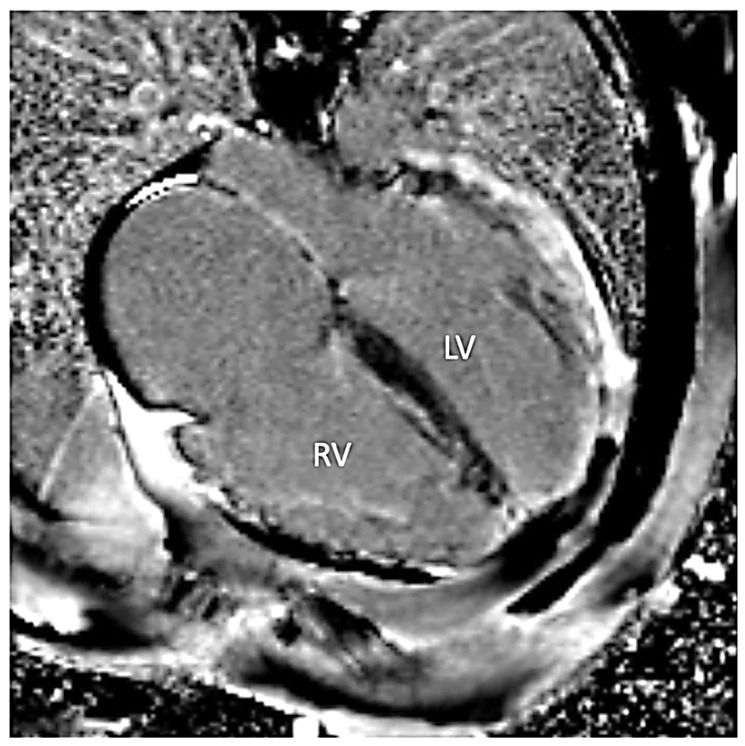
Late gadolinium enhancement four-chamber image demonstrating diffuse fibrosis in the left (LV) and the right ventricles (RV).

**Figure 5:**
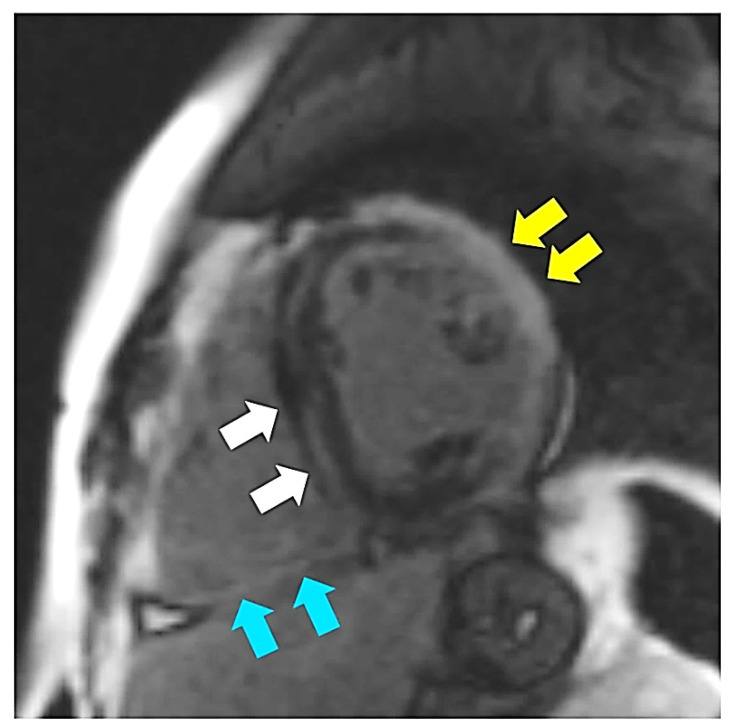
Late gadolinium enhancement short axis image demonstrating midwall fibrosis in the interventricular septum (white arrows) as well as transmural fibrosis in the left ventricular lateral wall (yellow arrows) and in the inferior wall of the right ventricle (blue arrows).

Since early childhood, he was under care of cardiologists with diagnosis of hypertrophic cardiomyopathy and dominant right ventricular cardiomyopathy. No significant ventricular arrhythmia was found in medical records of the patient. Since the patient reached the age of 30 years, symptoms and signs of biventricular heart failure were observed. Following an episode of paroxysmal atrial flutter with rapid ventricular response, the patient was treated with cardioversion that led to sinus arrest. With concomitant conduction disease (right bundle branch block and left anterior fascicular block), he had cardiac resynchronization therapy with defibrillator implanted for secondary prevention (at age of 31 years). At that time computed coronary angiography showed normal coronary arteries and he was referred for a genetic diagnosis with progressive heart failure symptoms of New York Heart Association (NYHA) class III. On standard 12-lead electrocardiogram sinus bradycardia 54/min with wandering atrial pacemaker, sinistrogram, left atrial fascicular block, and right bundle branch block were found (Figure 1). Serum creatine kinase activity was within the reference range (106 U/l). On cardiac imaging, mildly enlarged left ventricle with global left ventricular ejection fraction of 25–30% (Video S1 in the Supplementary Materials), increased myocardial thickness in the septal segments up to 14 mm and akinesis with wall thinning of lateral segments of the left ventricle were found (Figure 2 and Figure 3). In addition, enlarged right heart chambers with dominant right ventricular dilatation, severe diffuse hypokinesis of the right ventricle (Video S1, Figure 2 and Figure 3), and severe tricuspid insufficiency were observed. Diffuse late gadolinium enhancement consistent with myocardial fibrosis was present (Figure 4 and Figure 5). With recurrent exacerbations of heart failure, he was referred for orthotopic heart transplantation (OHT). On invasive assessment before OHT, there was no pulmonary hypertension 37/17/27mmHg and pulmonary arteriolar vascular resistance was 0.42 Wood units.

Successively, subsequent neurological and cardiac symptoms progressed, which are summarized in Table 1. In general, the most dominating neurological symptom was cerebellar ataxia, while the most significant cardiologic manifestation was severe biventricular heart failure due to mixed cardiomyopathy eventually leading to the successful cardiac transplantation at 33 years of age. Family history in this patient was negative.

All investigations were performed in accordance with relevant guidelines and regulations. The study was carried out following the rules of the Declaration of Helsinki of 1975.

Proband and his family members gave their informed consent for performance of diagnostic analyzes and procedures.

### 2.2. Laboratory Analyses

#### 2.2.1. Pathological Examination of Explanted Heart

Heart tissue samples came from the left and right ventricles, and interventricular septum. Tissue samples were formalin fixed and paraffin embedded for routine light microscopy in hematoxylin and eosin staining.

**Figure 6:**
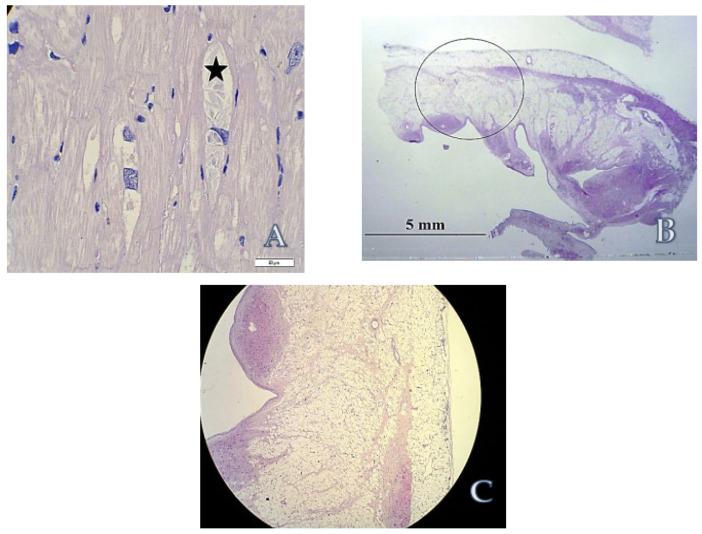
Light microscopy of the biopsy specimens obtained from explanted heart. Hematoxylin and eosin staining; (**A**) Histology of left ventricular wall, asterisk shows amorphous intravacuolar deposits, surrounding cardiomyocytes contain also typical lipofuscin granules. Original magnification: ×400. Scale bar: 50 μm. (**B**) Whole mount specimen of right ventricular wall showing ‘ARVC-like’ fatty replacement. Original magnification: ×5. (**C**) Close-up of the area encircled in (**B**). Original magnification: ×5.

Histopathological assessment of explanted heart revealed diverse, inconsistent results:

1. Numerous cardiomyocytes showed hypertrophy and perinuclear vacuolization containing amorphous deposits suggesting the possibility of metabolic disorder, possibly Fabry disease (Figure 6A). Normal alfa-galactosidase activity in peripheral blood leukocytes and plasma excluded this diagnosis.
2. The presence of remarkable thinning and fatty–fibrous replacement of right ventricular wall strongly suggested ARVC-like (resembling arrhythmogenic right ventricular cardiomyopathy) pattern (Figure 6B,C).

#### 2.2.2. Next Generation Sequencing (NGS)

**Figure 7:**
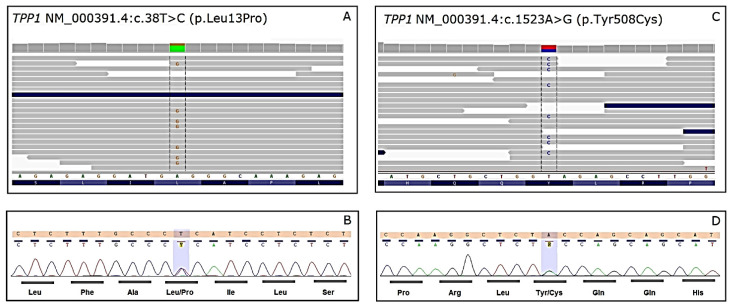
Compound heterozygous variants in *TPP1* gene in the proband. (**A**) NGS results: Integrative Genomics Viewer (IGV) view of *TPP1* NM_000391.4:c.38T > C (p.Leu13Pro) variant in the proband; (**B**) chromatogram from Sanger sequencing of NM_000391.4:c.38T > C (p.Leu13Pro) variant in the proband. (**C**) NGS result: IGV view of NM_000391.4:c.1523A > G (p.Tyr508Cys) variant in the proband. (**D**) Chromatogram from Sanger sequencing of NM_000391.4:c.1523A > G (p.Tyr508Cys) variant in the proband.

In order not to prolong the diagnostic odyssey, the NGS was performed with the use of TruSight One sequencing panel, which covers disease-associated regions of 4813 genes. Sanger sequencing was applied to confirm the findings of NGS and co-segregation of the variants in the family. The NGS sequencing run for proband achieved 30,794,778 reads with a mean value of 36.56. Above 73% and 95% of the target region was covered 20 and 10 times, respectively. Analysis of NGS data did not reveal genetic cause of cardiomyopathy in the patient. In the proband, we did not detect any rare (allele frequency ≤ 0.01 in gnomAD Genomes) variants with classification: VUS, Likely Pathogenic, or Pathogenic in coding regions of genes rated green from PanelApp (panel “Cardiomyopathies-including childhood onset” version v1.4 [19]. Notably, we did not detect any rare (frequency <0.01 in gnomAD) variants classified according to ACMG criteria as VUS, Likely Pathogenic, or Pathogenic in the *GLA* and *LAMP2* genes, so we ruled out Fabry and Danon diseases. Taking into account neurologic symptoms, obtained results revealed potentially pathogenic variants in two genes (Figure 7):

(a) *TPP1*—compound heterozygous—novel, likely pathogenic NM_000391.4: c.1523A > G, p.Tyr508Cys, rs769195711 variant, and previously reported NM_000391.4: c.38T > C, p.Leu13Pro variant classified according to The American College of Medical Genetics and Genomics (ACMG) as variant of uncertain significance (VUS) with minor pathogenic evidence [20,21,22]; Sanger sequencing of *TPP1* variants in proband’s parents confirms in trans inheritance: c.1523A > G.p.Tyr508Cys is of maternal origin and c.38T > C, p.Leu13Pro is of paternal origin (Table 2); In silico analysis of p.Tyr508Cys variant showed high pathogenicity scores in 11 (BayesDel addAF, DANN, DEOGEN2, EIGEN, FATHMM-MKL, LIST-S2, M-CAP, MVP, Mutation assessor, MutationTaster, SIFT) out of 12 algorithms (only PrimateAI algorithm scored p.Tyr508Cys variant as ‘Tolerated’). In silico analysis of p.Leu13Pro variant showed high pathogenicity scores in 6 (BayesDel addAF, DANN, M-CAP, MVP, MutationTaster, SIFT) out of 12 algorithms. Following algorithms: DEOGEN2, EIGEN, FATHMM-MKL, LIST-S2, Mutation assessor, PrimateAI) scored p.Leu13Pro variant as ‘Tolerated/Benign/Neutral/Low’.

(b) *ABCD1*—hemizygous novel missense mutation NM_000033.4:c.50G > A, p.Arg17His rs782693577, VUS according to ACMG classification (Table 2).

Mutations in the *ABCD1* gene are responsible for X-linked adrenoleukodystrophy/adrenomyeloneuropathy (X-ALD/AMN). To verify the pathogenicity of the novel mutation, the level of very long chain fatty acids (VLCFA) was analyzed in serum samples from the proband and his mother. Results excluded X-ALD/AMN, see Table 2.

Mutations in the *TPP1* gene, encoding the tripeptidyl peptidase 1 enzyme, are known to be causative for the late infantile neuronal ceroid lipofuscinosis 2 (CLN2). To verify the pathogenicity of the novel mutations found in proband, the activity of TPP1 was determined in peripheral blood leukocytes with the fluorescent methylcoumarine derivative as a substrate (Ala-Ala-Phe-7-amido-4-methylcoumarin; Sigma-Aldrich cat. no. A3401). The deficient activity of TPP1 in proband and normal in his parents confirmed the diagnosis. In the proband, residual TPP1 activity in leukocytes was 8.8 U/mg protein/h. This is about 27% of the lowest or 18% of the mean control values (reference range 30–81 U/mg protein/h; mean ± SD: 47.4 ± 10.7 U/mg protein/h, n = 101; N.B. the control enzyme—beta-galactosidase was in the reference range and did not indicate any failure to the leukocytes sample). In our laboratory, the mean TPP1 residual activity in leukocytes for CLN2 patients was 1.99 U/mg protein/h and it accounted for 6.6% of the lowest or 4% of mean control values (TPP1 activity range for CLN2 patients: 0.1–13.2 U/mg protein/h, n = 94). TPP1 activities measured in leukocytes from proband’s parents were 51 and 57 U/mg protein/h and they were within the reference range. In other CLN2 obligate heterozygotes TPP1 activity ranged from 24 to 42 U/mg protein/h (n = 7).

#### 2.2.3. Ultra-Structure Studies

In the next step of diagnostic process, we extended the analyses to include the transmission electron microscopy studies. For this purpose, the samples of the left ventricle of the explanted heart were taken from paraffin blocks. After deparaffination, the material was fixed in 2.5% glutaraldehyde and postfixed in 1% OsO_4_ and routinely processed to Spurr resin. Ultrathin sections were stained with uranyl acetate and lead citrate and examined under an Opton DPS 109 electron microscope.

**Figure 8:**
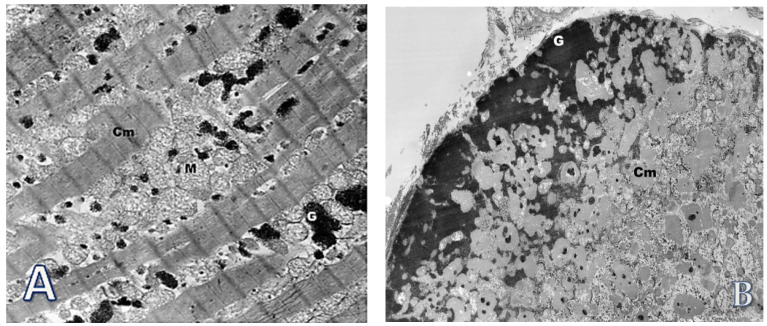

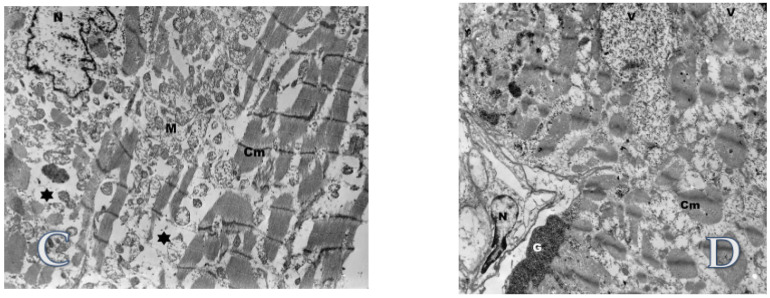
Transmission electron microscopy studies of the biopsy specimens obtained from explanted heart. (**A**) Cardiomyocyte (Cm) in longitudinal section. Numerous swollen mitochondria (M) and glycogen (G) in the sarcoplasm are visible. Original magnification: ×7000; (**B**) Cardiomyocyte (Cm) in cross section. Accumulation of glycogen (G) in an area with loss of myofibrils. Original magnification: ×3000; (**C**) A bizarrely shaped cell nucleus (N) in the sarcoplasm of the cardiomyocyte (CM). Multiple loss of myofilaments (asterisk) and accumulation of damaged mitochondria (M). Original magnification: ×4400; (**D**) Extensive loss of myofibrils in cardiomyocyte (Cm). The vacuoles (V) with dispersed glycogen are visible and also the accumulation of glycogen (G) near the sarcolemma. Nucleus (N). Original magnification: ×4400.

Subsequent ultra-structural analysis (Figure 8) of cardiomyocytes showed enlarged and bizarre shaped nuclei, extensive loss of myofibrils with formation of vacuoles, numerous swollen mitochondria, and accumulation of glycogen in sarcoplasm. There was no accumulation of lipofuscin as well as ultrastructural profiles of curvilinear or fingerprint bodies and granular osmiophilic deposits (GRODs) in lysosomes, characteristic of NCLs.

## 3. Discussion

*TPP1*, encoding the tripeptidyl peptidase 1 enzyme, is known as the causative gene for autosomal recessive late infantile neuronal ceroid lipofuscinosis disease 2 (CLN2 disease), which is characterized by epilepsy, loss of vision, ataxia, and a rapidly progressive course, leading to early death. Tissue electron microscopic studies typically reveal curvilinear bodies. Clinical manifestations in classic CLN2 display a continuum of disease severity presumably related to residual enzyme activity (from late infantile to juvenile CLN2 phenotype).

In 2013, Sun et al. identified *TPP1* as the causative gene for autosomal recessive spinocerebellar ataxia type 7 (SCAR7). To date, seven patients with SCAR7 from three families were reported [16,17], who showed ataxia and residual activity of TPP1, but no ophthalmologic abnormalities or epilepsy. Additionally, the slowly progressive evolution of the disease until old age and absence of ultrastructural curvilinear profiles differed from the known CLN2 phenotypes (Table 3).

It was suggested that loss of function variants abolishing TPP1 enzyme activity lead to CLN2 disease, whereas variants that diminish TPP1 enzyme activity lead to slowly progressive SCAR7 [16,17].

Our case of a 36-year-old male patient with TPP1 deficiency presents atypical CLN2/SCAR7 phenotype with cerebellar-extrapyramidal syndrome, dilated cardiomyopathy, and severe cerebellar atrophy in neuroimaging. A causal link between the TPP1 defect and the cardiac and CNS involvement in the patient seems very probable, although not fully confirmed at the functional level. Results of the NGS study did not show any other possible variants in genes associated with cardiomyopathies or ataxia.

Similarly to SCAR7 patients, he has residual TPP1 enzyme activity as well as the slow progression of the disease and absence of ultrastructural curvilinear profiles in electron microscopy. Unlike in SCAR7 patients, he presents with extrapyramidal signs and cardiomyopathy. In contrast to classic CLN2 and SCAR7, in our patient, the TPP1 deficiency was caused by missense mutations on both alleles (Table 3).

Lourenco et al. summarized that, in patients with atypical CLN2 disease, seizures, language abnormalities, and behavioral disorders were the first symptoms. Moreover, the median age of symptoms onset was 6 years, which is more than described for the classic late infantile form of CLN2 (2–4 years) [15].

In our patient, the first symptoms were observed at 4 years of age and included dysarthria and cardiac arrhythmia, indicating that this is a patient with signs of atypical form of CLN2 but also with cardiac disease. While the devastating effect of CLN2 upon the brain is well known, it should be emphasized that both peripheral and autonomic nervous system, as well as other somatic tissues, are also injured [23]. Still, non-obvious is the involvement of cardiac dysfunction in neuronal ceroid lipofuscinoses, most prominently seen in CLN3 disease [23].

A term of mixed cardiomyopathy, defined when a cardiomyopathy had features of more than one functional type is often used in pediatric population [24]. Our patient had segmental hypertrophy, segmental thinning, and global diffuse biventricular hypokinesis with more pronounced right heart involvement. Of interest, among 26 known causes of mixed cardiomyopathies in the study by Cox et al., approximately one-fourth (26.9%) are related to inborn errors of metabolism [24]. Moreover, this type of storage disease in our patient was associated with lack of pulmonary hypertension and lack of ventricular arrhythmia. In contrast, in a patient with glycogenosis type IV there was no decrease in right ventricular function, severe pulmonary hypertension, and common complex ventricular arrhythmia [25].

Cardiac involvement in CLN3 is characterized by repolarization disturbances, ventricular hypertrophy, and sinus-node dysfunction, ultimately leading to severe bradycardia and/or other conduction abnormalities, starting in the mid-teens [26].

Cardiac complications have also been described in CLN1, CLN2, and CLN6 patients. In CLN1, patients’ cardiac symptoms were not detected during their lifetime, but in a post-mortem examination mild dilation of the ventricles and accumulation of storage material were noted. Two CLN6 patients from one family developed arrhythmia and conduction blocks. In post-mortem analysis of their hearts, fibrosis, accumulation of auto fluorescent storage material, and curvilinear bodies were seen [27,28].

So far, one CLN2 patient was described with atypically slow disease progression, who developed right and left anterior bundle branch blocks and episodic bradycardia at 23 years of age. Several episodes of supraventricular tachycardia manifested at 23 and 27 years of age. In addition, a transient second-degree atrioventricular conduction block also emerged at 27 years of age. Atrial fibrillation and aggravation of the atrioventricular conduction block resulted in progressive bradycardia and cardiac death at the age of 28 years [29]. Cardiac pathological changes, covering cardiomyopathy and repolarization disturbances, constitute a potential anesthetic risk to both young and older CLN patients [29].

Severe cardiac impairment was developed in a canine model of CLN2, accompanied by hepatic disturbances, after treatment with exogenous TPP1 enzyme administered through the intra-cerebroventricular injection route alone, indicating a potential need for systemic administration of TPP1 [30].

According to Guelbert et al., cardiac anomalies and conduction disorders can be caused by the storage of lipopigments found not only in the neurons but also in other tissues, including the heart [31]. Opposite to this, the curvilinear or fingerprint bodies were absent in heart sample from our patient. However, it cannot be excluded that in biopsy samples taken from other tissues (or organs) such storage would be confirmed. Fealey et al. claim that pathologic findings involved in cardiac impairment consist of mild to moderate myocyte hypertrophy, myocardial deposition of lipopigments (particularly in the conduction system), and variable chamber enlargement. The only consistent feature among patients with neuronal ceroid lipofuscinoses and those affected with cardiac disorder are arrhythmic activity or repolarization abnormality [32].

Indeed, the most consistent feature of the heart involvement due to ceroid lipofuscinosis is cardiac conduction system disease, with abundant accumulation of lipopigments and degeneration seen in all components of the conduction system, clinically presenting from sinus bradycardia [27,33,34] to atrio-ventricular block of varying degree and bundle branch blocks [27,29,33,35]. Patient reported here also presented with advanced conduction disease (RBBB +LAFB, and third degree a-v block). In NCL patients supraventicular arrhythmias (supraventricular tachycardia, atrial fibrillation) and ventricular tachycardias were noted. These disturbances were also seen in our proband.

This is the first case of end stage dilated cardiomyopathy leading to severe heart failure and consequent heart transplantation. However, there are some data showing more pronounced cardiac muscle involvement in NCLs.

Sakajiri et al. showed cardiac autopsy findings in two cases with adult type ceroid lipofuscinosis who died because of arrhythmia [27]. Of note, the heart showed—apart from a slight increase in the accumulation of the lipofuscin-like lipopigments in the myocardial fibers—also slight to severe fibrosis and infiltration of fat cells in the myocardium, similarly as in our patient, in whom fat infiltration of the right ventricle was most prominent (CMR, autopsy data), thus resembling arrythmogenic right ventricular cardiomyopathy.

On the other hand, Hofman et al. showed in two patients with juvenile type NCL that the storage was associated with hypertrophy and dilation of both ventricles, which is consistent with clinical diagnosis of mixed cardiomyopathy, presenting both features of dilated and hypertrophic cardiomyopathies [34].

Classic hypertrophic cardiomyopathy may lead to heart failure through depression of the contractility, thinning of the myocardial wall and development of end stage dilated cardiomyopathy.

We suppose that the processes of ‘hypertrophy’ (due to accumulation of storage material) and thinning (due to replacement fibrosis) in the storage diseases are simultaneous, as continuous supply of the storage material takes place throughout life and leads to death of the overloaded cardiomyocytes. In hypertrophic cardiomyopathy, the phase of dilation follows the phase of hypertrophy. That is why it is difficult to define the type of cardiomyopathy in our patient, who presented both features of hypertrophic, arrhythmogenic right ventricular, and end-stage dilated cardiomyopathies.

Ultra-structure analysis of the explanted heart from our patient revealed enlarged and bizarre shaped nuclei, extensive loss of myofibrils with formation of vacuoles, numerous swollen mitochondria, and accumulation of glycogen in sarcoplasm of cardiomyocytes. Danon disease, as well as other glycogenosis, have been ruled out after whole exome sequencing. Interestingly, this picture resembles findings described in heart tissue samples from patients with dilated cardiomyopathy of unknown origin, in which changes observed in electron microscopy consisted also of enlargement and varying shape of nuclei, numerous very small mitochondria, proliferation of T tubules, and accumulation of lipid droplets and glycogen. Lipofuscin, myelin figures, and vacuoles were numerous. The most prominent alteration was the loss of contractile elements resulting in large cellular areas filled with non-specified cytoplasm containing only mitochondria, ribosomes, and glycogen [36].

## 4. Conclusions

In summary, a patient described here as affected with CLN2/SCAR7 is the first case presenting with cardiac manifestations before 14 years of age. This report also emphasizes that TPP1 deficiency should be considered in the differential diagnosis of early-onset cerebellar ataxia and dilated cardiomyopathy of unknown origin.

## Figures and Tables

**Table 1:** Development of neurologic and cardiac symptoms in the presented patient.

Age	Symptoms
	NEUROLOGIC
4 years	dysarthria (speech disturbances and stuttering)
22 years	ataxia, mild right hand muscle weakness, and tremor
28 years	MRI of the brain—severe cerebellar atrophy and moderate cortical and subcortical atrophy
32 years	- slow progression of the neurological symptoms—dysarthria, ataxia, dystonia, and tremor of the right hand - psychological assessment—slightly decreased mental acuity - CT of the brain—severe cerebellar atrophy - VEP examination—bilateral slowed neural conduction in the visual pathways - BAEP examination—bilateral hearing impairment
	CARDIOLOGIC
4 years	cardiac arrhythmia (paroxysmal supraventricular tachycardia (SVT) and unclassified cardiomyopathy)
30 years	- Cardiac conduction system disease (right bundle branch block, RBBB; and left anterior fascicular block, LAFB) - mild symptoms of biventricular heart failure
32 years	- paroxysmal supraventricular tachycardia and paroxysmal atrial flutter - dilated cardiomyopathy with heart failure - implantation of CRT defibrillator capable of cardiac resynchronization therapy (CRT-D)
33 years	successful cardiac transplantation

BAEP—brainstem auditory evoked potentials; CRT—cardiac resynchronization therapy; CRT-D—CRT with cardioverter-defibrillator; CT—computed tomography; LAFB—left anterior fascicular block; MRI—magnetic resonance imaging; RBBB—right bundle branch block; SVT—supraventricular tachycardia; VEP—visual evoked potentials.

**Table 2:** Laboratory findings in proband and his parents.

Analysis	Proband	Mother	Father	Reference Range
Explanted heart tissue—light microscope	- severe myocyte hypertrophy - in the cells: perinuclear vacuoli - zation with inclusions - ARVC-like lesion	n.d.	n.d.	-
Explanted heart tissue—transmission electron microscopy (ultrastructure cell examination)	- enlarged and bizarre shaped nuclei - extensive loss of myofibrils with formation of vacuoles and/or intermyofibrillar spaces - accumulation of glycogen granules in sarcoplasm - increased amount of mitochondria - absence of lipofuscin accumulation as well as of ultrastructural curvilinear profiles, fingerprint bodies, and granular osmiophilic deposits (GRODs) in lysosomes	n.d.	n.d.	-
Alfa-galactosidase	- plasma: 5.8 nmol/mL/h - blood leukocytes: 12.4 nmol/mg protein/h	n.d.	n.d.	- plasma: 8.6 ± 1.5 - blood leukocytes: 10 ± 2.5
NGS—TruSight One sequencing panel: *TPP1* gene *ABCD1* gene	p.Leu13Pro, VUS with minor pathogenic evidence (PM2, PP2) /p.Tyr508Cys, likely pathogenic (PM1, PM2, PP2, PP3) p.Arg17His, VUS (PP2, PP3, BS2)	p.Tyr508Cys p.Arg17His	p.Leu13Pro -	One novel and one previously reported variants Novel variant
VLCFA in serum	C24:0/C22:0 = 0.898 C26:0/C22:0 = 0.010	C24:0/C22:0 = 0.847 C26:0/C22:0 = 0.011	n.d.	<0.960 <0.030
TPP1 activity in blood leukocytes	8.8 U/mg protein/h	51	57	47.4 ± 10.7 range 30–81

n.d.—not done; ARVC—arrhythmogenic right ventricular cardiomyopathy; NGS—next generation sequencing; VLCFA—very long chain fatty acids.

**Table 3:** Clinical and laboratory features of classic CLN2 (late infantile and juvenile), SCAR7, and our patient [16,17 with modifications].

Clinical and Laboratory Features	CLN2 Late Infantile	CLN2 Juvenile	SCAR7 (7 Patients)	Our Patient
General	very severely affected	less severely affected	mild phenotype and protracted course	mild phenotype and protracted course
Age of onset	2–4 years	6–10 years	childhood or teenage	childhood
Age of death	5–15 years	12–40 years	> 60 years	alive at 37
Clinical findings	seizures, dementia, visual loss	seizures, dementia, visual loss, ataxia	cerebellar ataxia, pyramidal signs/no, deep sensory loss	cerebellar ataxia, tremor, dystonia, cardiomyopathy
Neuroimaging	cerebral atrophy	cerebral atrophy	cerebral atrophy	cerebral atrophy
TPP1 enzyme activity	extremely low/none	residual/very low	residual	residual
Ultra-structure features (EM)	curvilinear bodies	curvilinear bodies/ GROD/ fingerprint profiles	none/ GROD/ fingerprint profiles	none
*TPP1* gene variants (alleles)	Many (null/null)	Many (null/partial affected)	nonsense/missense (null/minor modification)	missense/missense

## Data Availability

The data presented in this study are available upon request from the corresponding author. The data are not publicly available due to personal data protection.

## Supplementary Materials

The following are available online at https://www.mdpi.com/article/10.3390/life12010003/s1, Video S1: Steady-state free precession four-chamber cine demonstrating severe wall motion abnormalities of the left and the right ventricle.
