# Supplementary material for: Tripeptidyl Peptidase 1 (TPP1) Deficiency in a 36-Year-Old Patient with Cerebellar-Extrapyramidal Syndrome and Dilated Cardiomyopathy
Source: Life (Basel). 2021 Dec 21;12(1):3. doi: 10.3390/life12010003 (PMC8779458; doi:10.3390/life12010003)
Supplement: Supplementary file 1 [file life-12-00003-s001.zip › life-1406269 - Supplementary.pdf]

life

MDPI

Supplementary material of Tripeptidyl Peptidase 1 (TPP1) Deficiency in a 36-Year-Old patient with Cerebellar-Extrapyramidal Syndrome and Dilated Cardiomyopathy

Video S1: Steady-state free precession four-chamber cine demonstrating severe wall motion abnormalities of the left and the right ventricle.
